# Predictive equations for respiratory muscle strength according to international and Brazilian guidelines

**DOI:** 10.1590/bjpt-rbf.2014.0044

**Published:** 2014

**Authors:** Isabela M. B. S. Pessoa, Miguel Houri, Dayane Montemezzo, Luisa A. M. Silva, Armèle Dornelas De Andrade, Verônica F. Parreira

**Affiliations:** 1 Departamento de Fisioterapia, Pontifíca Universidade Católica de Minas Gerais (PUC/Minas), Belo Horizonte, MG, Brazil; 2 Departamento de Zootecnia, Universidade Federal de Minas Gerais (UFMG), Belo Horizonte, MG, Brazil; 3 Programa de Pós-graduação em Ciências da Reabilitação, UFMG, Belo Horizonte, MG, Brazil; 4 Fisioterapeuta, Belo Horizonte, MG, Brazil; 5 Departamento de Fisioterapia, Universidade Federal de Pernambuco (UFPE), Recife, PE, Brazil; 6 Departamento de Fisioterapia, UFMG, Belo Horizonte, MG, Brazil

**Keywords:** maximum respiratory pressures, reference values, rehabilitation, physical therapy, MIP, MEP

## Abstract

**Background:**

The maximum static respiratory pressures, namely the maximum inspiratory pressure (MIP) and maximum expiratory pressure (MEP), reflect the strength of the respiratory muscles. These measures are simple, non-invasive, and have established diagnostic and prognostic value. This study is the first to examine the maximum respiratory pressures within the Brazilian population according to the recommendations proposed by the American Thoracic Society and European Respiratory Society (ATS/ERS) and the Brazilian Thoracic Association (SBPT).

**Objective:**

To establish reference equations, mean values, and lower limits of normality for MIP and MEP for each age group and sex, as recommended by the ATS/ERS and SBPT.

**Method:**

We recruited 134 Brazilians living in Belo Horizonte, MG, Brazil, aged 20-89 years, with a normal pulmonary function test and a body mass index within the normal range. We used a digital manometer that operationalized the variable maximum average pressure (MIP/MEP). At least five tests were performed for both MIP and MEP to take into account a possible learning effect.

**Results:**

We evaluated 74 women and 60 men. The equations were as follows: MIP=63.27-0.55 (age)+17.96 (gender)+0.58 (weight), r^2 ^of 34% and MEP= - 61.41+2.29 (age) - 0.03(age^2^)+33.72 (gender)+1.40 (waist), r^2^of 49%.

**Conclusion:**

In clinical practice, these equations could be used to calculate the predicted values of MIP and MEP for the Brazilian population.

## Introduction

The maximum static respiratory pressures (MRPs), namely the maximum inspiratory (MIP) and expiratory pressure (MEP), reflect the strength of the respiratory muscles^1^^-^ 3. As these measures are simple, non-invasive, and have diagnostic and prognostic value, several authors have established reference values for populations of diverse ethnicities^4^^-^ 9. 

There is large inter-subject variability in MRP values. The biological characteristics of populations, measurement technique, and equipment used all contribute to this variability^7^^-^ 13. The variation in MRPs and the need to standardize evaluation procedures has led the *American Thoracic Society, European Respiratory Society *(ATS/ERS)^2^ and the Brazilian ThoracicAssociation (Sociedade Brasileira de Pneumologia e Tisiologia -SBPT)^3^ to publish guidelines for the testing of respiratory muscles, including the performance of MRP measurements. 

For testing, International and Brazilian recommendations suggest the use of a diver's type mouthpiece; the presence of a drain hole approximately 2 mm in internal diameter; the performance of the test by an experienced operator, who must stimulate the subject to perform a MIP against an occluded airway and a MEP against an occluded area near or within residual volume (RV) and within total lung capacity (TLC), respectively; adopting a sitting posture; instruction prior to the maneuver and encouragement during the maneuver; prevention of air leaks around the mouthpiece, directing the subject to hold the cheeks with the hands during expiratory pressure and to press the lips tightly around the mouthpiece; and, recording the maximum mean pressure (MMP). 

ATS/ERS^2 ^stresses the importance of using digital instruments to ensure the validity of the measurements used to determine MMP (maximum mean pressure sustained for 1 second). International^2^and Brazilian^3 ^guidelines have similar recommendations, although there are points on which they differ, such as the use of a nose clip (SBPT recommendation) and the maximum number of tests for each measurement. SBPT^3 ^recommends a maximum of five tests and considers the learning effect of the repeated measurements, allowing the performance of more tests if the last value was the highest. 

In Brazil, after publication of the guidelines, two studies were published proposing reference values for MRPs^8^^,^ 13. The methodological recommendations proposed by the scientific societies were not satisfied in their entirety in either study^2^^,^ 3. 

In this context, the aim of this study was to establish MRP reference values, considering the ATS/ ERS^2^ and SBPT^3^ recommendations, for a sample of adults in a population from Belo Horizonte, MG, Brazil. 

## Method

### Sample

Sample size calculation was performed according to Simões et al.'s study^13^. A level of statistical significance of 5%, a power of 90% and 20% estimated effect were adopted for the calculations^13^^,^ 14. Effect size (Cohen d) was based on calculations of the differences between the mean MIP and MEP in men and women for each age group, as used in Simões et al.'s study^13^, yielding a sample size of 117 individuals. Subsequently, the required number of individuals of each gender in each age group was estimated so that the sample would be representative, based on analysis of the Brazilian Institute of Geography and Statistics (Instituto Brasileiro de Geografia e Estatística -IBGE) - 2010 census for the population of Belo Horizonte, MG, Brazil^15^. 

The non-probabilistic sample consisted of volunteers of both genders, selected from the community by personal invitation, telephone call, or electronic communication, who fulfilled all of the study's inclusion criteria. The inclusion criteria were as follows: healthy adults between 20 and 89 years, with spirometric parameters within the limits predicted for the Brazilian population^16^and body mass index (BMI) within the normal range (18.5 Kg/m^2^≤BMI≤29.9 Kg/m^2^)^17^. The exclusion criteria were as follows: history of current smoking; exposure to occupational environmental risk; reported history of neuromuscular, respiratory, and/or heart disease; cognitive impairment (in participants aged over 60 years); fever in the previous three weeks and/or cold and/or sinus infection in the week before the test; use of drugs such as oral steroids, central nervous system depressants, barbiturates, and/or muscle relaxants; exhaustive exercise in the 48 hours preceding the test; absence of dental elements; limiting muscle pain in the upper limbs; resting blood pressure (BP) greater than or equal to 160/110 mmHg^18 ^and/or hemoglobin saturation (SpO_2_) less than 90% and/or heart rate (HR) greater than 85% of maximum heart rate before execution of the maneuvers; and inability to understand and/or perform the procedures in the research protocol. The test was interrupted if there were reports of respiratory and/or muscular discomfort during performance. 

The study was approved by the Ethics Committee of the Universidade Federal de Minas Gerais (UFMG), Belo Horizonte, MG, Brazil (CAAC Protocol 0425.0.203.000-10), and all participants signed the terms of free and informed consent. 

### Measuring instruments

### Digital Manometer

A digital manometer (NEPEB-LabCare/UFMG) was used to measure MRP^19^^,^ 20, in which the pressures were measured by pressure transducers with an operating range of 500 cmH_2_O^19^. A diver's mouthpiece with a 2 mm diameter hole and nose clip were used to measure MRP^2^^,^ 3 ^,^ 21. The manometer was calibrated every six months, as established by Ferreira et al.^19^.

### Spirometer

*Vitalograph *(*Vitalograph 2120, Ennis, Ireland*) and *Pony* (*Pony FX* ^(r)^ *, Cosmed, Rome, Italy*) were used, and acceptability and reproducibility criteria were adopted according to SBPT standards^22^. Spirometric data were derived from the forced vital capacity (FVC) maneuver and interpreted in accordance with the values predicted by Pereira et al.^16^. 

### Electrical bioimpedance

*Quantum III BIA - 101Q RJL-101 (Detroit, USA*) equipment was used for the measurement of lean and fat mass. The instrument was a quadrupole model with digital display, as per recommendations^23^.

### Mechanical scale, portable digital scale and stadiometer

A Filizola analog scale (Filizola Ind. Ltda, São Paulo, Brazil) was used to measure weight and height. The scale was coupled to a stadiometer with resolutions of 100 g and 0.5 cm, which was calibrated prior to each measurement. A 100 g resolution portable digital scale (*Life Electronic Scale, Geratherm* ^(r)^ *, *Germany) and a 0.1 cm resolution portable stadiometer (Alturexata, Belo Horizonte, Brazil) were used for evaluations conducted outside the University. To evaluate the reliability of the scale measures, the weight of ten volunteers was measured on the two scales at random. No significant difference was observed between the data obtained on the two scales (67.22±10.2 Kg *versus *66.86±10.0 Kg, p=0.14, intraclass correlation coefficient 0.99). These data were used to calculate BMI. 

### Waist circumference and waist-hip ratio

An anthropometric tape measure (metric) consisting of an inelastic material was used. The standing position was adopted with arms at the volunteer's sides, feet together, and relaxed abdomen^17^. The waist circumference measurement was taken by firmly applying the tape in the trunk region between the lower edge of the last rib and the upper edge of the iliac crest^17^. Hip measurement was performed by applying the tape firmly to the maximum posterior extension of the buttocks^17^. 

### Fitness

Volunteers were asked about their level of physical activity and professional occupation to classify them as active or sedentary, as recommended by the American College of Sports Medicine (ACSM)^24^: active (weekly caloric expenditure above 450 MET-min/week) or sedentary (below 450 MET-min/ week), ascertained by self-report^25^. 

### Procedures

The initial evaluation included the following data: personal data; measuring weight, height, and waist and hip circumferences; vital data -PA (*Littman Classic, St. Paul, USA* stethoscope and *Tycos, NY, USA *sphygmomanometer); HR and SpO_2 _(*Nonim, USA *pulse oximeter); and physical fitness by self-report^24^^,^ 25. The Mini Mental State Exam was also applied for elderly volunteers and a cutoff of 23/24 adopted^26^. Electrical bioimpedance was then measured, after which the volunteers were offered a standardized food intake. 

The pulmonary function test was then performed. Later, after standing for at least 10 minutes, the subjects' MRP measurement was taken randomly (electronic randomization). All procedures were performed on a single visit and by a single evaluator. 

For MRP measurements (i.e. MMP), the subjects remained in a sitting position with legs and trunks supported, with instructions and a demonstration being given prior to the tests^2^^,^ 3. All MRP measurements were performed using the nose clip^3^. To measure MIP, the participants performed two to three breaths at functional residual capacity (FRC) level and then were requested to perform an expiration to RV, indicating that time by elevating the participant's own hand^3^. At this point, the participant was encouraged to generate maximal inspiratory pressure, and simultaneously, the examiner proceeded to close the occlusion orifice and give a standardized verbal command^3^^,^ 20. 

The same procedure was performed to measure MEP, except for the final verbal instruction, which was to request an inspiration to TLC, followed by encouragement to perform maximal expiratory pressure^2^^,^ 3 ^,^ 20. To measure MEP, the researcher pressed the cheeks of the volunteers to prevent air leakage^2^. 

The minimum operating time was 1.5 seconds so that the maximum sustained pressure could be observed for 1 second^2^. All subjects underwent at least five repetitions of each test, with a 1-minute interval between them. The pressure measurement was considered complete when the participant performed three acceptable maneuvers (no air leaks between the lips and/or nose clip and at least 1.5 second duration)^2^^,^ 3 and when, of these tests, there were three reproducible ones (one with variation less than or equal to 10% and the other with a variation of no more than 20% with pressure of higher value)^2^^,^ 3. The largest measure could not be the last test because of a potential learning effect^3^. The highest MMP value was selected^2^. 

### Data reduction

The MRPs were operationalized by *Manovac *software version 4.1, using the MMP variable^20^^,^ 21 ^,^ 27. The formula LLN = value predicted by the regression equation formula -(1.645 x standard error of the estimate) was used to obtain the lower limit of normality (LLN)^3^.

### Statistical analysis

Descriptive data statistical analysis (means and dispersions) was performed, and the normal distribution and homogeneity of variance of the variables were ascertained using the Lilliefors and Cochran & Bartletti tests, respectively. Once the nature of responses was established in their parametric or non-parametric condition (Pearson, Spearman, and Kendall), a correlation matrix was established of the independent variables (i.e. gender, age, height, weight, BMI, waist circumference, waist-hip ratio, and physical activity level) between each pair of these variables and with the measured responses (i.e. MIP, MEP). Using the *backward stepwise *system, non-significant independent variables (p>0.05) were disregarded, noting the continued determination capacity (r^2^) of the model. After the explanatory effects of each model were defined, they were tested for each of their quadratic effects as well as for possible interactions. All analyses were processed using the *Statistical Package for Social Sciences *program (SPSS 15.0, *Chicago, IL, USA*) and the *Statistical Analysis System *(SAS 12.0, *Carey, NC, USA*). 

## Results

One hundred sixty-four individuals were initially evaluated. Of these individuals, 20 were not included because they were obese (BMI above 30 Kg/m^2^), and two were excluded due to being underweight (BMI below 18.5 Kg/m^2^). Eight subjects were excluded due to obstructive or restrictive disorders being identified during pulmonary function testing. The final sample consisted of 134 volunteers. All volunteers completed the proposed protocol. The mean age was 47±18 years for women and 43±16 years for men. The final sample comprised 51% sedentary women and 39% sedentary men. The percentage of body mass was evaluated in a subgroup of individuals (n=64), verifying that the men had a mean lean body mass percentage of 72.8±4.7 Kg and the women of 64.4±5.0 Kg. Participants presented the following spirometric data: forced vital capacity (FVC): women=94.1±10.9% of predicted value and men 92.5±10.0% of predicted value; forced expiratory volume in 1 second (FEV_1_): women=93.8±11.2% of predicted value and men=92.4±9.5% predicted value); and FEV_1_/FVC ratio (%): women=81.5±5.7 and men=81.6±4.7. 

Table 1 shows the anthropometric and demographic characteristics of the volunteers distributed by gender across five age subgroups. 

**Table 1:** Demographic and anthropometric characteristics of 134 individuals according to sex and age subgroups.

Groups	N	Age (years)	Height (m)	Weight (Kg)	BMI	FBM (%)	LBM (%)	W-C (cm)	W-H	Physical fitness
Females										
20-29	16	24 (3)	1.63 (0.06)	59 (7)	22 (2)	34.3 (3.6)	65.7 (3.6)	70.9 (4.7)	0.71 (0.04)	68%
30-39	14	36 (3)	1.64 (0.06)	61 (10)	23 (3)	36.3 (5.3)	63.7 (5.3)	74.1 (8.7)	0.76 (0.05)	57%
40-49	14	44 (4)	1.63 (0.07)	63 (7)	24 (2)	38.0 (4.7)	62.0 (4.7)	78.1 (6.0)	0.79 (0.04)	43%
50-59	11	56 (3)	1.58 (0.07)	63 (10)	25 (2)	41.0 (1.5)	59.1 (1.5)	79.1 (5.8)	0.81 (0.05)	45%
>60	19	71 (8)	1.53 (0.05)	59 (8)	25 (3)	35.0 (6.2)	65.0 (6.2)	79.6 (8.6)	0.81 (0.06)	42%
Total	74	47(18)	1.60 (0.07)	61 (8)	24 (3)	35.6 (4.6)	64.4 (5.0)	76.4 (7.7)	0.78 (0.06)	51%
Males										
20-29	15	24 (3)	1.77 (0.06)	73 (9)	23 (2)	24.3 (3.7)	75.7 (3.7)	80.1 (4.2)	0.83 (0.04)	40%
30-39	14	34 (3)	1.76 (0.09)	83 (12)	27 (2)	28.7 (5.6)	71.3 (5.6)	89.4 (6.4)	0.87 (0.05)	50%
40-49	11	44 (3)	1.73 (0.06)	76 (9)	25 (2)	25.6 (3.1)	74.4 (3.1)	90.6 (5.8)	0.97 (0.20)	27%
50-59	10	53 (3)	1.72 (0.08)	78 (7)	27 (2)	27.7 (5.2)	72.3 (5.2)	93.8 (5.4)	0.95 (0.05)	40%
>60	10	69 (8)	1.69 (0.07)	76 (7)	27 (2)	29.4 (4.6)	70.6 (4.6)	95.7 (7.0)	0.98 (0.07)	40%
Total	60	43(16)	1.74 (0.08)	77 (9)	25 (2)	27.2 (4.7)	72.8 (4.7)	89.2 (7.8)	0.91 (0.11)	39%

Data presented as mean and standard deviation (+/-)

N =sample

BMI =body mass index

FBM =fat body mass

LBM =lean body mass

W-C =waist circumference

W-H =waist hip ratio

Physical fitness=percentage sedentary.

Table 2 presents the mean MRP values with their coefficients of variation. The mean MIP and MEP values were 24% and 33% lower in women than in men, respectively. 

**Table 2:** Values of maximal respiratory pressures obtained for each subgroup with the coefficient of variation.

FEMALES (N=74)			MALES (N=60)	
Ages (Year)	MIP (cmH_2_O)	MEP (cmH_2_O)	MIP (cmH_2_O)	MEP (cmH_2_O)
20-29	99.4 (20.7)	114.2 (23.1)	126.1 (21.7)	144.5 (20.5)
30-39	99.0 (6.4)	121.8 (33.0)	126.1 (32.9)	178.7 (38.4)
40-49	97.9 (26.5)	121.9 (34.1)	132.6 (31.3)	163.5 (39.0)
50-59	87.7 (24.0)	119.4 (35.3)	118.9 (50.9)	212.9 (21.2)
>60	74.8 (16.4)	91.8 (29.6)	98.5 (12.6)	155.4 (50.5)
Total	91.1 (26.1)	112.1 (32.2)	121.3 (30.7)	167.4 (40.12)
CV (%)	29	29	25	24

Data presented as mean and standard deviation (+/-).

N =sample

MIP =maximal inspiratory pressure

MEP =maximal expiratory pressure

CV =coefficient of variation

Table 3 shows the prediction equations for the MRPs. The following variables in each MIP regression equation were considered: age (p<0.0001), gender (p=0.0047) and weight (p=0.0245), explaining 34% of MIP variation; and for MEP: gender (p=0.0004), age^2 ^(p=0.0071), age (p=0.0516), waist circumference (p=0.0125), explaining 49% of MEP variation. 

**Table 3:** Predictive equations for maximal respiratory pressures.

Predictive equations	r^2^	SEE
**MIP** (cmH_2_O)=63.27–0.55 (age)+17.96 (sex)+0.58 (weight)	34	26.3
**MEP** (cmH_2_O)=–61.41+2.29 (age)–0.03 (age^2^)+33.72 (sex)+1.40 (waist)	49	32.8

MIP =maximal inspiratory pressure

MEP =maximum expiratory pressure

**waist:** =waist circumference in cm

weight in Kg

r2 =coefficient of determination

SEE =standard error of the estimate

For females, the constant is multiplied by zero (sex=0). For males, the constant remains (sex=1). To calculate the lower limit of normality: Mean-1.645 (standard error of the estimate). To calculate the upper limit of normality: Mean+1.645 (standard error of the estimate).

## Discussion

To our knowledge, this work was the first Brazilian study to establish MRP reference values as set forth in the methodological recommendations proposed by ATS/ERS^2^ and by the SBPT^3^that used digital equipment, which provides highly accurate and valid measures. 

This study was conducted with the aim of building prediction models for MRPs in a sample of the adult population of Belo Horizonte, MG, Brazil according to the methodological recommendations of ATS/ERS^2 ^and SBPT^3^. The issue of the existence of several benchmarks for MIP measures was confirmed in a recently published systematic review^28^, in which the low methodological quality of the existing articles was noted, contributing to an appreciation of the difficulty, in research and especially in clinical situations, of choosing an appropriate prediction equation. 

One reason for the variability in MRP values is a lack of methodological standardization among studies. After the ATS/ERS publication^2 ^on methodological recommendations for MRP testing, two Brazilian studies were published^8^^,^ 13 that did not fully comply with the recommendations, the main deviation being the non-use of a digital instrument that would enable the operationalization of MMP. It is known that valid measures provide correct interpretations of the respiratory muscle strength rates, which are essential for the diagnosis of respiratory muscle weakness. All Brazilian studies have used an analog manometer, compromising measurement accuracy. This study therefore has the advantage/originality of building prediction equations from MRPs created from a digital manometer. 

In studies on benchmarks, the premise that the sample must be healthy and representative of the population for which the study was conducted is valid. This definition has been ignored in certain studies^8^^,^ 13 in terms of the absence of a report on a pulmonary function test^8 ^and the fact that the sample consisted entirely of sedentary individuals^13^. In contrast to all other studies on reference values, this study took the additional care to apply the Mini Mental State Exam for elderly subjects (i.e. over 60 years) to verify the absence of cognitive impairment, as the test was volitional and could have been affected by the understanding, cooperation, and coordination of the individual^2^. 

In addition to a lack of procedural standardization, variability in reference values may also be explained by the influence of individual (biological) factors involved in the study sample^29^. In this study, it was found that when analyzed by gender, the mean MRP values showed significant variation between subjects in the same age group (26 to 37% of MIP, Table 2). A high coefficient of variation was also reported in studies by Hautmann et al.^7 ^and Enright et al.^30 ^(25 to 27% and 32 to 39%, respectively). Such variations could be attributed to the degree of motivation and cooperation of the volunteer^5^; to the value of the elastic recoil pressure of the respiratory system^5^; coordination during the test^10 ^and the degree of individual activation of musculature during the test^10^; the intrinsic differences of individual muscles^7^, such as speed of muscle contraction^1^; and genetic and environmental factors^5^. 

Surprisingly high MEP values were observed in men aged 50 to 59 when compared to lower age groups. Importantly, there were a significant number of active and motivated individuals tested in this age group. These subjects met all inclusion criteria, so there was no justification for their exclusion from the analysis. 

All studies on reference values evaluate the predictive power of physical characteristics on MRPs, and there appears to be a weak association between them, as evidenced by the low coefficients of determination. There is no consensus on the influence of certain individual factors (e.g. height, weight, age) on the MRPs of men and women. Height has been found to be a positive predictor^31^, a negative one^9^^,^ 32, or not a predictor of MIP in women^8^^,^ 12 ^,^ 13 and a negative predictor in men^6^. 

There is consensus that gender is the best predictor of MRPs^4^^,^ 7 ^,^ 8 ^,^ 10 ^,^ 11. Values for MIP and MEP were, on average, 24% and 33% higher in men compared to women, which is consistent with the study by Simões et al.^13^. Some authors claim that respiratory muscles behave like skeletal muscles^10^^,^ 12 ^,^ 30 ^,^ 33, so strength is proportional to the sectional area of the muscle^34^. In this study, analysis of body composition was performed in 64 individuals of both genders, and it was found that the mean lean body mass percentage (i.e. muscle) was higher in men, one possible explanation for the difference in MRP between genders. 

The results of this study showed that age significantly influenced MRP, being a negative predictor, which was consistent with the three previous Brazilian studies^8^^,^ 12 ^,^ 13. The effect of age on MRPs in both genders is still questionable^4^^,^ 5 ^,^ 11 ^,^ 35, although most studies reported a decrease in MRPs with advancing age in both genders, especially for MIP^7^^,^ 8 ^,^ 10 ^-^ 13. The cutoff point for the decline in MIP differed between studies, ranging from 30^10 ^to 65^7 ^years. This difference may prevent the appearance of a negative correlation with age as a result of the age group studied, as in the study of Camelo et al.^36^, which analyzed the 20-49 year age group. The low number of subjects above 55 years of age also explains the absence of a negative correlation between MRPs and age in certain studies^4^^,^ 35. One possible explanation for MIP decreasing with advancing age is the aging process, which causes increased RV and decreased inspiratory capacity^37^. 

MEP showed a quadratic relationship with age. This finding is consistent with previous studies^10^^,^ 38. Possible explanations for the decrease in MEP are the loss of elastic recoil of the chest cavity, the presence of calcification in joints, and increased thoracic kyphosis, given that all these factors contribute to low rib cage compliance and decreased MEP, which is performed based on TLC^37^. Atrophy, decreased metabolic efficiency, and a decline in nerve conduction velocity may also explain the decrease in MRPs with advancing age^10^^,^ 30. 

Weight was a positive predictor for MIP, a finding in agreement with previous studies^9^^,^ 30 ^,^ 32 ^,^ 39, in particular Simões et al.'s study^13^. There is no consensus regarding the variation of MRPs due to weight gain^10^^,^ 13. Both pulmonary function and respiratory muscle strength improved with a small increase in body weight, called the "muscularity effect," as there is a theory that relates the weight and length of different isometric muscle groups^40^. In this study, both weight and muscle percentage correlated positively with one another and each in isolation with MIP. It can be hypothesized that the influence of weight on MIP is related to the higher percentage of lean mass of the respiratory muscles. 

Waist circumference was a positive predictor of MEP. This study was the first in which this variable was considered in the model for MEP. The volunteers in this study were not obese and had normal mean waist circumference values. It is possible that the positive correlation with MEP was due to the larger abdominal muscle. It could be hypothesized that there is a cutoff point for decline in MEP similar to the behavior of the MIP when it is correlated with waist circumference (MIP declines from 95-105 cm in waist circumference, according to Carpenter et al.^29^). Future studies using a sample with increased circumference values might investigate this hypothesis more thoroughly to establish the type of predictive model that exists between waist circumference and MEP and thereby explain the influence of visceral fat on abdominal muscle strength. 

A limitation of this study was the small sample sizes in the different age subgroups, in particular, the over 70-year-old group. Sample size directly affects the predictive accuracy of the equation^41^. However, representativeness of the sample was achieved in terms of the number of individuals of each gender and in age group being in keeping with IBGE data for the population of Belo Horizonte, MG, Brazil. 

In conclusion, this Brazilian study, conducted with a sample of the population of Minas Gerais (Belo Horizonte, MG, Brazil), provides equations, means, and standard deviations by age group, as well as formulas for calculating the lower and upper limits of normality. The study is innovative in that it strictly follows the methodology proposed by 

Brazilian and international standards, notably the use of a digital manometer to ensure the validity of measurements. 
